# Translation of a tissue epigenetic signature to circulating free DNA suggests *BCAT1* as a potential noninvasive diagnostic biomarker for lung cancer

**DOI:** 10.1186/s13148-022-01334-3

**Published:** 2022-09-19

**Authors:** Cora Palanca-Ballester, David Hervas, Maria Villalba, Teresa Valdes-Sanchez, Diana Garcia, Maria Isabel Alcoriza-Balaguer, Marta Benet, Raquel Martinez-Tomas, Andres Briones-Gomez, Jose Galbis-Caravajal, Alfonso Calvo, Oscar Juan, Agustin Lahoz, Enrique Cases, Juan Sandoval

**Affiliations:** 1 Biomarkers and Precision Medicine Unit, Health Research Institute La Fe, Av. Fernando Abril Martorell, 106, 46026 Valencia, Spain; 2 Department of Applied Statistics and Operational Research and Quality, Universitat Politècnica de València, Valencia, Spain; 3 CIBERONC, ISCIII, 28029 Madrid, Spain; 4 IDISNA and Program in Solid Tumors, Center for Applied Medical Research (CIMA) and Department of Pathology, Anatomy and Physiology, School of Medicine, University of Navarra, Pamplona, Spain; 5 BEMYGENE, Parc Científic University of Valencia, Paterna, Spain; 6 Epigenomics Unit, Health Research Institute La Fe, Valencia, Spain; 7 Pneumology Service, University Hospital La Fe, Valencia, Spain; 8 Thoracic Surgery Service, University Hospital La Ribera, Alzira, Spain

**Keywords:** Epigenetics, DNA methylation, ddPCR, Plasma, Circulating DNA, *BCAT1*, Lung cancer, Noninvasive

## Abstract

Lung cancer patients are diagnosed at late stages when curative treatments are no longer possible; thus, molecular biomarkers for noninvasive detection are urgently needed. In this sense, we previously identified and validated an epigenetic 4-gene signature that yielded a high diagnostic performance in tissue and invasive pulmonary fluids. We analyzed DNA methylation levels using the ultrasensitive digital droplet PCR in noninvasive samples in a cohort of 83 patients. We demonstrated that *BCAT1* is the candidate that achieves high diagnostic efficacy in circulating DNA derived from plasma (area under the curve: 0.85). Impact of potentially confounding variables was also explored.

**Supplementary Information:**

The online version contains supplementary material available at 10.1186/s13148-022-01334-3.

## Introduction

Lung cancer (LC) is currently the leading cause of cancer-related death worldwide accounting for approximately a third of all cancer diagnosed and deaths. LC is one of the most aggressive tumor types, with a 5-year survival rate that remains consistently low, not exceeding 31% [1]. Several factors are associated with the poor outcome of LC patients. One of them is late diagnosis. Only 16% of the cases are diagnosed at early stages due to the relative lack of symptoms or signs, and consequently, approximately two-thirds of LCs are detected at advanced stages of the disease. By that time, the options for effective therapeutic intervention are limited and the survival rates drop significantly.

Noninvasive detection appears to be a key factor in increasing LC patient survival. Thus, an increased interest has raised to the development of imaging techniques and molecular biomarkers. In screening strategies, low-dose computed tomography has shown a significant reduction in LC mortality in randomized trials. However, there are some open questions and areas of optimization which require further efforts and studies to accomplish a complete and worldwide implementation in the clinics [2].

Genetic alterations are fundamental to define cancer types. However, cancer behavior depends as well on changes in gene expression. Therefore, a current intense line of research lies on gene expression regulatory events such as epigenetic factors. These epigenetic changes occur early in cancer cells, with possible implication in the complete set of processes defined as the hallmarks of human cancers. Hence, epigenetic biomarkers, mainly DNA methylation, have been shown to play an important role in carcinogenesis at an early stage. Thus, epigenetic biomarkers are emerging as a promising approach to improve clinical management, including cancer diagnosis [3].

Different epigenetic candidates have been proposed based on two strategies: single gene hypothesis-driven or unbiased data-driven studies. Based on this first approach, *SHOX2* hypermethylation is the most widely studied epigenetic alteration. It was first reported by Schmidt et al. using bronchial fluid aspirates [4] and further studies have continued evaluating its diagnostic performance in other fluids. Regarding the second approach, our group in 2016, took advantage of a high throughput epigenomic strategy using Infinium beadchips to identify in lung tissues a novel 4-gene epigenetic signature (*BCAT1*, *CDO1*, *ZNF177* and *TRIM58*) for early detection in LC. The results from our epigenetic signature presented high diagnostic accuracy and were validated in large and independent cohorts of FFPE tissue and minimally invasive samples such as bronchial fluid aspirates and induced sputa. Moreover, it provided a balanced and flexible approach able to cater to both extreme scenarios: the high sensitivity and low specificity of low-dose CT in screening programs and the high specificity and low sensitivity of cytology. It is worth to mention that this signature obtained better diagnostic performance than the standard pathologic assessment of cytologic specimens, especially in peripherally located tumors and the implementation of clinical predictive tools, such as nomograms, increase the individualized risk assessment for patients [5].

Precision medicine implementation demands the use of robust epigenetic diagnostic biomarkers in noninvasive samples, mainly blood or plasma. In recent years, circulating tumor DNA (ctDNA) present in the plasma of patients, included in the concept of “liquid biopsy,” is being considered as a promising strategy for biomarker cancer detection and is now attracting a huge interest [6]. Therefore, in this study, we have extended the knowledge of this previously identified tissue epigenetic signature and assessed its diagnostic potential in plasma-derived circulating free DNA (cfDNA) (liquid biopsy), using the ultrasensitive and quantitative methylation-specific PCR (digital droplet PCR). This study provides a potential novel diagnostic epigenetic candidate in blood, *BCAT1*, as one of the most suitable noninvasive biomarkers that may help to improve the timing and accuracy of LC diagnosis.

## Materials and methods

### Study samples

#### Patients

The study population included 83 recruited individuals, 44 patients with non-small cell lung cancer (NSCLC) and 39 non-neoplastic patients with pulmonary disease, from the University Hospital La Fe and Hospital la Ribera in Spain. Non-cancer patients were followed up during the duration of the study to confirm that they did not develop cancer. Descriptors of the patients for each single case are shown in Additional file 1: Table S1. Blood samples were collected in PAXgene® Blood ccfDNA Tube (Qiagen) and centrifuged at 1900*g*, 10 min at 4 °C. Plasma was stored carefully at −80 °C until further processing.

#### Cell lines

A549, H209, H520 cell lines were obtained from the American Type Culture Collection (Manassas, VA) and used for primer optimization. We selected one cell line with high DNA methylation (A549 for all four genes) and others with low DNA methylation (H520 for *BCAT1* and H209 for the rest three genes) in the CpG of interest, based on our database using the Infinium DNA methylation array applied to cell lines. The cell lines were tested by certified third party laboratories for authenticity (STR assay) and tested for the absence of mycoplasma.

### ccfDNA isolation and bisulfite conversion

DNA from cell lines was isolated with the QIAamp DNA kit (Qiagen, Germantown, MD, USA) and used to optimize the primers of ddPCR. cfDNA from human plasmas were isolated with the QIAamp Circulating Nucleic Acid (Qiagen, Germantown, MD, USA), following the protocols provided by the manufacturer, and used to test the methylation level of *BCAT1* in both groups: NSCLC patient and control human samples. The bisulfite conversion was carried out in cfDNA (up to 50 ng) with the EZ-DNA Methylation-Lightning Kit (Zymo Research, Irvine, CA, USA). Bisulfite-treated DNA was eluted in 30 μl of elution buffer and stored at − 80 °C until further processing.

### Digital droplet PCR analysis

For ddPCR, specific primers to identify either the methylated (labeled with FAM) or the unmethylated (labeled with HEX) CpGs to amplify were synthesized (Additional file 2: Table S2). Primers for ddPCR were designed according to Bio-Rad recommendations (http://www.bio-rad.com). The QX200 Droplet Generator (Bio-Rad, Hercules, CA, USA) was used before DNA amplification with the following conditions: 95 °C for 10 min; 40 cycles of 94 °C for 30 s and 55 °C for 1 min; 98 °C for 10 min. The optimal annealing temperature was chosen after performing a temperature gradient assay for *BCAT1*, *CDO1* and *ZNF177* primer sets in DNA isolated from cell lines. Trim58 region was unable to be amplified. DNA amplification was carried out with the C1000 Touch Thermal Cycler (Bio-Rad). After the PCR, the QuantasoftTM software (Bio-Rad) was used for the analysis, using the RED (Rare Event Detection) option.

### Statistical analysis

Data were summarized using mean and range in the case of continuous variables and relative and absolute frequencies in the case of categorical variables. Discrimination capacity of BCAT1 was assessed by determining ROC curves and AUC values, as well as by testing for associations between methylation values and group (either control or LC) in a multivariable logistic regression model which included age, sex and smoking status as covariables. In addition, to test differences between mean methylation values of tumor versus non-tumor samples, the Wilcoxon–Mann–Whitney test was applied. All statistical analyses were performed using R (version 4.1.2) and R package pROC (version 1.18.0).

## Results

The clinical cohort included 83 human plasma samples from 44 NSCLC patients and 39 control cancer-free patients and the clinical characteristics are shown in Table 1. Groups were comparable in terms of mean age and gender proportions, but there was a slight imbalance regarding smoking status. The two most frequent NSCLC subtypes (adenocarcinoma and squamous cell carcinomas) were also represented in this cohort. Control patients were individuals with a lung-related pathology, who did not show any histologic evidence of tumoral malignancy.

**Table 1:** Clinical characteristics of patients with lung cancer and tumor-free individuals (controls) with respiratory diseases

Patients	Discovery cohort	Non-tumoral donor (*n* = 39)
	Lung cancer patients (*n* = 44)	
Age (years)	68 (53–82)	64 (31–90)
Sex		
Male	34 (77%)	25 (64%)
Female	10 (23%)	14 (36%)
Smoking history		
Smoker	22 (50%)	6 (15%)
Former smoker	9 (20%)	17 (44%)
Nonsmoker	13 (30%)	14 (36%)
Unknown	0 (0%)	2 (5%)
Stage		
I	4 (9%)	
II	7 (16%)	
III	3 (7%)	
IV	30 (68%)	
Histology		
Adenocarcinoma (AC)	31 (70%)	
Squamous cell carcinoma (SCC)	13 (30%)	

Data are average (range) or number (%)

**Fig. 1:**
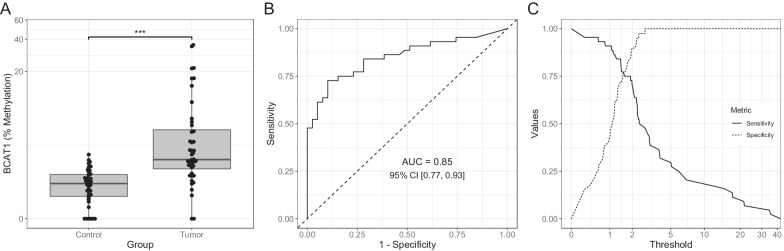
BCAT1 DNA methylation levels in plasma samples using digital droplet PCR. **A** DNA methylation levels in plasma from patients with lung cancer and control donors. *p* values for the analyses were calculated using the multivariable logistic regression model. ***p < 0.001. **B** ROC curves and areas under the curve (AUC). **C** sensitivity and specificity profiles for the different possible cutoff values of the results

In the case of TRIM58, three different probe sets were tested, but all of them were unable to amplify the target sequence (data not shown). Therefore, this gene was discarded from the analysis. Then, we analyzed the cfDNA methylation ratio that reflected the percentage of methylated alleles of the correspondent CpG for the three genes remaining in the epigenetic model (*BCAT1*, *CDO1* and *ZNF177*). For *ZNF177*, although the primers were optimized in cell lines, the results clearly indicated poor technical quality in patients (Additional file 3: Fig. S1). Comparative analysis of CDO1 showed no significant differences between NSCLC and controls (Additional file 4: Fig. S2). However, *BCAT1* DNA methylation levels were significantly higher (*p* < 0.001) in tumor samples as compared with non-tumoral controls (Fig. 1A). When used as a biomarker for the discrimination between tumor/control samples, *BCAT1* showed notable accuracy, with an area under the ROC curve (AUC) of 0.85 (Fig. 1B). After adjusting for age, sex and smoking status in a multivariable logistic regression model, higher BCAT1 values were associated with a significant increase in the risk of having NSCLC (adjusted OR = 3.11, 95% CI [1.72, 6.79], *p* < 0.001). To test BCAT1 performance in early stages, a parallel logistic regression model, which excluded the stage IV NSCLC patients and included all the same covariables as the main model. This analysis revealed that higher BCAT1 values were associated with a significant increase in the risk of having cancer (adjusted OR = 2.36, 95% CI [1.18, 5.71], *p* = 0.031). Since methylation values are continuous within the range from 0 to 1, a sensitivity–specificity profile was generated for the different possible cutoff values (Fig. 1C). The cutoff that maximizes both sensitivity and specificity was a methylation value of 1.98%, with a sensitivity of 0.73 and a specificity of 0.90. However, a cutoff methylation value of 1.42% yielded a higher sensitivity value of 0.84, associated with a lower, but still adequate specificity of 0.72.

## Discussion

Late diagnosis is one of the major reasons associated with high mortality in LC. Current detection methods based on low-dose computed tomography and minimally invasive cytology show low positive predictive value and low sensitivity, respectively. Therefore, there is an urgent necessity to implement molecular noninvasive biomarkers to improve LC detection and prolong survival. This study was designed to transfer and evaluate the utility of our previously identified epigenetic signature in lung tumoral tissue and bronchial aspirates into blood samples. We used ddPCR to quantify DNA methylation ratio of the 3 remaining genes in cfDNA, but only *BCAT1* showed significant and robust results. It is worth stressing that the methylation status of *BCAT1* alone determined in blood yielded a notable discrimination capacity (AUC = 0.85), with sensitivity and specificity profiles comparable to those obtained in our previous study using the 4-gene signature in bronchial fluids (AUC = 0.91) [5].

Several studies have been published in plasma samples, reporting differentially methylated genes when comparing LC patients versus control donors [7]. The seminal study reported by Kneip et al. in 2011 validated the diagnostic performance of *SHOX2*, which showed an AUC = 0.78 using real-time PCR [8]. Later, the analysis of a combination of *SHOX2* and *PTEGR4* methylation levels in blood demonstrated significant discriminatory performance in distinguishing patients with LC from subjects without malignancy (AUC = from 0.86) [9]. Other genes have been found to be differentially methylated in plasma samples when comparing LC patients and healthy controls, including *RASSF1A* and *RARB2* [10] or an epigenetic signature as an adjunct to low-dose CT scan screening [11]. Our study takes advantage of the ultrasensitive technique ddPCR, instead of qPCR, with the advantage of evaluating one single biomarker with high AUC value.

Recently, Chen et al. reported an approach based on methylation microarrays and whole genome bisulfite sequencing (WGBS) directly in cfDNA, which identified an epigenetic signature, called PanSeer, for cancer detection [12]. Despite these promising epigenomic results, the implementation in the clinic might be a long and costly process. Therefore, the evaluation of reduced candidate genes, such as *BCAT1*, may currently be a more feasible and affordable strategy for noninvasive detection of LC.

This study presents some limitations, despite the 4 genes *BCAT1, CDO1, ZNF177* and *TRIM58* being promising candidates in our previous study, we were unable to amplify TRIM58 by ddPCR, and CDO1 and ZNF177 showed poor performance. In the case of *TRIM58,* we believe, that being located the CpGs of interest in a very high-density CpG island, involves that designing probes in this type of regions may be quite difficult and challenging. We expect the development of new probe design tools, specific for ddPCR, to overcome these difficulties. Furthermore, despite the excellent performance of *BCAT1* in stages I–III, we are aware that the number of early stage samples in our cohort is low. We also included smoking status as a covariate in our logistic regression model. This was motivated by a previous meta-analysis study in bibliography reporting an association between cigarette smoking and DNA methylation in 1405 genes, including BCAT1 [13]. Therefore, a future study using a large cohort in a prospective screening would be helpful.

In conclusion, our study suggests *BCAT1* as a potential noninvasive epigenetic biomarker for LC detection and might also be very helpful to monitor therapeutic efficacy or to define more precise screening programs. However, future clinical trials and validation studies in other laboratories with larger cohorts of patients should be carried out. Furthermore, combination studies to test potential synergistic effects among BCAT1 and other lung cancer biomarkers, such as SHOX2, PTEGR4, could also be considered.

## Supplementary Information

**Additional file 1: Table S1. **Clinical diagnostic of samples.

**Additional file 2: Table S2. **Assays primers and conditions.

**Additional file 3: Fig. S1.** ZNF177 DNA methylation levels in plasma samples using digital droplet PCR. DNA methylation levels in plasma from patients with lung cancer and control donors.

**Additional file 4: Fig. S2.** CDO1 DNA methylation levels in plasma samples using digital droplet PCR. DNA methylation levels in plasma from patients with lung cancer and control donors. *p* values for the analyses were calculated using the two-sided Mann–Whitney U test (not significant).

## Data Availability

Data are available from the corresponding author.

## Abbreviations

LC: Lung cancer
ctDNA: Circulating tumor DNA
cfDNA: Circulating free DNA
ddPCR: Droplet digital PCR
NSCLC: Non-small cell lung cancer
ROC: Receiver operating characteristic
AUC: Area under the curve
