# Supplementary material for: Translation of a tissue epigenetic signature to circulating free DNA suggests BCAT1 as a potential noninvasive diagnostic biomarker for lung cancer
Source: Clin Epigenetics. 2022 Sep 19;14:116. doi: 10.1186/s13148-022-01334-3 (PMC9487112; doi:10.1186/s13148-022-01334-3)
Supplement: Supplementary file 1 — Additional file 1: Table S1. Clinical diagnostic of samples. [file 13148_2022_1334_MOESM1_ESM.docx]

Supplementary table 1. Clinical diagnostic of samples

| Sample | Lung cancer patients (T) nontumoral donor (C) | Diagnostic | Included (I) or discarded (D) samples |
| --- | --- | --- | --- |
| 1 | T | AC | I |
| 2 | T | AC | I |
| 3 | T | AC | I |
| 4 | C | Acute respiratory infection | I |
| 5 | C | Emphysema and COPD | I |
| 6 | C | Asthma and pneumonia | I |
| 7 | T | AC | I |
| 8 | T | AC | I |
| 9 | C | Carcinoid tumor (benign) | I |
| 10 | T | AC | I |
| 11 | C | Pulmonary thromboembolism | I |
| 12 | T | AC | I |
| 13 | T | SCC | I |
| 14 | T | SCC | I |
| 15 | C | Respiratory infection | I |
| 16 | T | AC | I |
| 17 | T | SCC | I |
| 18 | T | AC | I |
| 19 | T | AC | I |
| 20 | C | Pneumonia | I |
| 21 | T | SCC | I |
| 22 | C | Hemoptysis and non-neoplastic node | I |
| 23 | T | AC | I |
| 24 | T | AC | I |
| 25 | T | SCC | I |
| 26 | T | AC | I |
| 27 | T | AC | I |
| 28 | T | AC | I |
| 29 | C | COPD and respiratory failure | I |
| 30 | T | SCC | I |
| 31 | C | Respiratory failure and bronchiectasis | I |
| 32 | T | AC | I |
| 33 | C | Interstitial pulmonary disease | I |
| 34 | T | SCC | I |
| 35 | T | AC | I |
| 36 | C | Pulmonary fibrosis | I |
| 37 | C | Bronchiectasis | I |
| 38 | T | AC | I |
| 39 | T | AC | I |
| 40 | C | Asthma | I |
| 41 | C | Benign lung nodule | I |
| 42 | T | AC | I |
| 43 | C | COPD and pulmonary infection | I |
| 44 | T | SCC | I |
| 45 | C | Bronchiectasis and hemoptysis | I |
| 46 | T | AC | I |
| 47 | C | Sarcoidosis | I |
| 48 | T | AC | I |
| 49 | C | Pulmonary fibrosis | I |
| 50 | T | AC | I |
| 51 | C | COPD and pulmonary emphysema | I |
| 52 | C | Asthma and pneumonia | I |
| 53 | T | AC | I |
| 54 | C | Eosinophilic bronchitis | I |
| 55 | T | AC | I |
| 56 | C | Bronchiectasis | I |
| 57 | T | AC | I |
| 58 | C | Hemoptysis and COPD | I |
| 59 | T | SCC | I |
| 60 | C | Bronchitis | I |
| 61 | C | Hemoptysis | I |
| 62 | T | AC |  |
| 63 | C | Pulmonary emphysema and pneumonia | I |
| 64 | T | AC | I |
| 65 | C | COPD | I |
| 66 | T | AC | I |
| 67 | C | Pulmonary silicosis | I |
| 68 | T | SCC | I |
| 69 | C | Bronchiectasis | I |
| 70 | T | AC | I |
| 71 | C | Pneumonia | I |
| 72 | T | AC | I |
| 73 | C | Langerhans cell histiocytosis and COPD | I |
| 74 | T | SCC | I |
| 75 | C | Dyspnea | I |
| 76 | T | AC | I |
| 77 | T | SCC | I |
| 78 | C | Respiratory infection | I |
| 79 | C | Interalveolar disease | I |
| 80 | C | Dyspnea and carcinoid tumor (benign) | I |
| 81 | C | Dyspnea | I |
| 82 | C | Acute respiratory infection | I |
| 83 | C | Carcinoid tumor (benign) | I |

Abbreviations: COPD (Chronic obstructive pulmonary disease), AC (Adenocarcinoma) and SCC (Squamous cell carcinoma).
