# Supplementary material for: Translation of a tissue epigenetic signature to circulating free DNA suggests BCAT1 as a potential noninvasive diagnostic biomarker for lung cancer
Source: Clin Epigenetics. 2022 Sep 19;14:116. doi: 10.1186/s13148-022-01334-3 (PMC9487112; doi:10.1186/s13148-022-01334-3)
Supplement: Supplementary file 2 — Additional file 2: Table S2. Assays primers and conditions. [file 13148_2022_1334_MOESM2_ESM.docx]

Supplementary Table 2: Assays primers and conditions

| **Digital Droplet PCR** | **Type** | **5'→3' or reference** | **Assay Annealing temperature** |
| --- | --- | --- | --- |
| **BCAT1** | Forward | TTTTAAGGGATGTTGGAG | 55 |
|  | Reverese | AACTAACCATAAAAAAACTAC |  |
|  | Probe methylated | TTTCGTGTTATTGTCGTTTT-FAM |  |
|  | Probe NO methylated | TTTGTGTTATTGTTGTTTTTTG-HEX |  |
| **CDO1** | Forward | TAAAGTGGGGGAGAGATTG |  |
|  | Reverese | ATACACACACAAATCAAATTCAA | 55 |
|  | Probe methylated | TGCGCGTGAGTCGTGTT- FAM |  |
|  | Probe NO methylated | ATGTGTGTGTGAGTTGTGTT- HEX |  |
| **ZNF177** | Forward | TTTAAGGGAGTAGGAGTATTTG |  |
|  | Reverese | ACAACCCTTTCTCAACTACA | 55 |
|  | Probe methylated | GTGGGCGTTCGTCGTTT- FAM |  |
|  | Probe NO methylated | AAGTGGGTGTTTGTTGTTT- HEX |  |
| **TRIM58** | Forward | TGTGTTTGGATTTTTTGTAGG |  |
|  | Reverese | CTCTCCACCAAACCC | Tested several |
|  | Probe methylated | TGCGAGAAGTCGGACGG- FAM |  |
|  | Probe NO methylated | TTTGTGAGAAGTTGGATGG- HEX |  |
