# Supplementary figures and images for: Translation of a tissue epigenetic signature to circulating free DNA suggests BCAT1 as a potential noninvasive diagnostic biomarker for lung cancer
Source: Clin Epigenetics. 2022 Sep 19;14:116. doi: 10.1186/s13148-022-01334-3 (PMC9487112; doi:10.1186/s13148-022-01334-3)

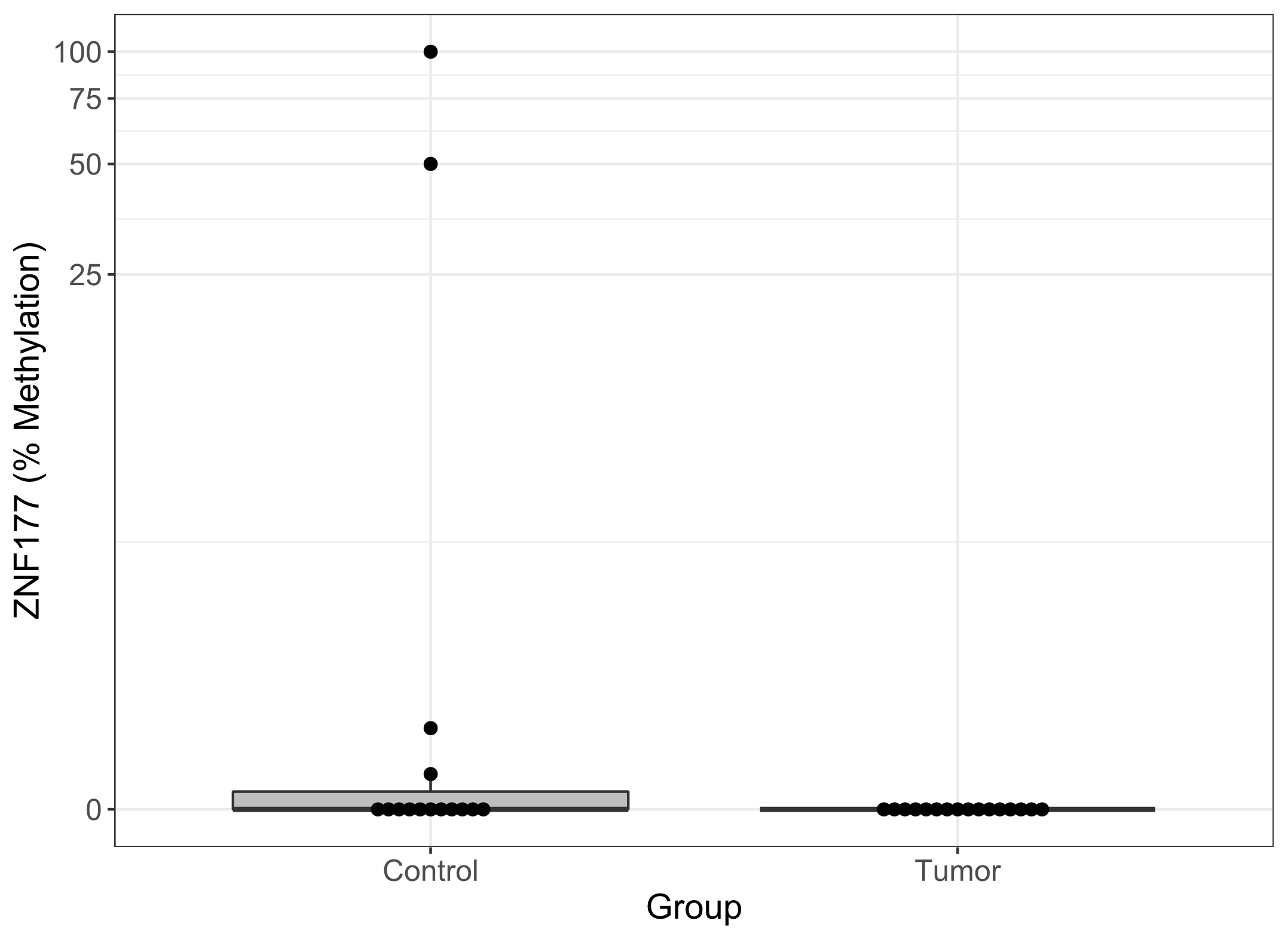

Supplement: Supplementary file 3 — Additional file 3: Fig. S1. ZNF177 DNA methylation levels in plasma samples using digital droplet PCR. DNA methylation levels in plasma from patients with lung cancer and control donors. [file 13148_2022_1334_MOESM3_ESM.jpg]

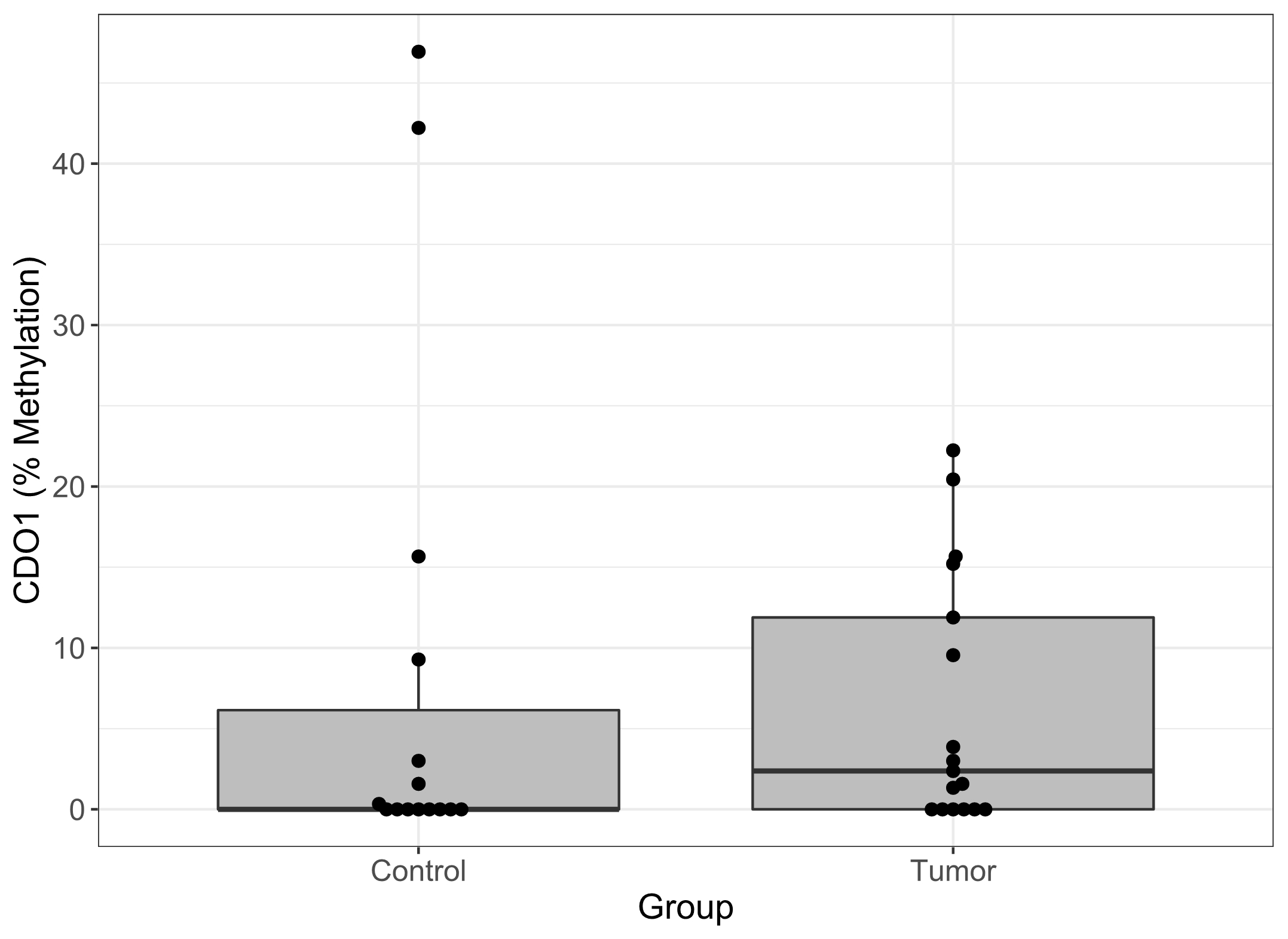

Supplement: Supplementary file 4 — Additional file 4: Fig. S2. CDO1 DNA methylation levels in plasma samples using digital droplet PCR. DNA methylation levels in plasma from patients with lung cancer and control donors. p values for the analyses were calculated using the two-sided Mann–Whitney U test (not significant). [file 13148_2022_1334_MOESM4_ESM.jpg]
